# Glioma-related epilepsy in patients with newly diagnosed and recurrent glioblastoma, IDH-wildtype: a study under the 2021 WHO CNS tumor classification

**DOI:** 10.1186/s12885-025-15351-x

**Published:** 2025-12-05

**Authors:** Xing Fan, Jianli Dai, Jiajia Liu, Gan You, Ke Li, Shengyu Fang, Jiahan Dong, Jiawei Shi, Jiangwei Wang

**Affiliations:** 1 Beijing Neurosurgical Institute, Capital Medical University, No. 119 South Fourth Ring West Road, Beijing, Fengtai District 10070 China; 2 Department of Neurosurgery, Beijing Tiantan Hospital, Capital Medical University, Beijing, 100070 China; 3 Institute of New Materials and Advanced Manufacturing, Beijing Academy of Science and Technology, Beijing, 100089 China

**Keywords:** Glioblastoma, Glioma-related epilepsy, Survival outcomes, Seizure control

## Abstract

**Background:**

The 2021 classification of central nervous system tumors significantly alters the defined category of glioblastoma (GBM). This study aims to evaluate the clinical relevance of glioma-related epilepsy (GRE) in patients with the newly classified GBM, IDH-wildtype.

**Methods:**

A single-center retrospective cohort study was performed. The correlation of GRE with clinicopathological features was explored via appropriate inter-group statistical methods. Kaplan-Meier analysis was employed to assess the prognostic value of preoperative GRE with respect to overall survival (OS) and progression-free survival (PFS). Multivariate binary logistic regression analysis was carried out to identify potential risk factors associated with inadequate seizure control.

**Results:**

The final cohort included 294 patients. Preoperative GRE was observed in 22.4% of newly diagnosed and 19.7% of recurrent GBM cases. In patients with newly diagnosed GBM, GRE was significantly associated with younger age (*p* < 0.001). Moreover, survival analysis confirmed the prognostic significance of preoperative GRE for improved OS and PFS (*p* = 0.012 and 0.004, respectively) in the same population. Moreover, in newly diagnosed GBM cases, preoperative GRE was identified as the independent risk factor for inadequate postoperative seizure control (OR 4.009, 95% CI 1.447–11.106, *p* = 0.008).

**Conclusion:**

The current study comprehensively describes the clinical correlations of GRE in GBM, IDH-wildtype. The incidence of preoperative GRE in GBM patients is approximately 20%. Among patients with newly diagnosed GBM, IDH-wildtype, preoperative GRE is more likely to occur in younger individuals and is associated with prolonged survival outcomes. Furthermore, a history of preoperative GRE may increase the risk of inadequate postoperative seizure control in this population.

## Background

Glioma is the most common and lethal type of primary cancer in the central nervous system (CNS), affecting approximately 6.0 cases per 100,000 people worldwide [1]. In the adult population, glioblastomas (GBMs), along with astrocytic and oligodendroglial tumors, constitute the majority of diagnosed gliomas in clinical neuro-oncology practice [2]. These three types are collectively categorized as adult diffuse gliomas, which have long raised significant concerns for neurooncologists and neurosurgeons due to their high incidence and dismal prognosis [3]. In recent decades, substantial progress has been made in glioma diagnosis and treatment. The development and application of integrated diagnosis, surgical assistance technologies, targeted therapies, and tumor-treating fields have greatly improved patient management and extended their survival [4, 5]. In this context, increasing attention has been directed toward improving the quality of life of glioma patients, and glioma-related epilepsy (GRE) has gained scholarly attention as a major comorbidity of gliomas.

Adult diffuse gliomas are highly epileptogenic, and the incidence of GRE in patients varies from 40 to 90% according to tumor pathology and grade [6]. Tumors with IDH mutations, oligodendroglial components, or low-grade histology are generally more likely to induce GRE [7]. The volatile and unpredictable nature of GRE can considerably weaken the independent viability and social capability of patients and simultaneously add to their anxiety and depression [8, 9]. Furthermore, GRE is often challenging to control, even in those who have received appropriate anti-tumor treatments [10]. A better understanding of GRE can potentially offer valuable insights that could enhance the overall management of glioma patients. However, the glioma classification system has evolved dramatically over the past decade, rendering many prior interpretations of GRE outdated according to the 2021 World Health Organization (WHO) classification of CNS tumors [4]. Consequently, further research on GRE in the context of the updated glioma classification framework is critically needed.

In the updated classification, one of the most notable changes is the rebranding of GBM as “GBM, IDH-wildtype”, which reflects revolutionary modifications in its diagnostic criteria and biological understanding. As a result, elucidating the characteristics of GRE within the newly defined GBM is of great importance, while the relevant clinical evidence is still quite limited at present. In the current study, we conducted a comprehensive analysis of the clinical correlates of GRE in newly diagnosed and recurrent GBM, IDH-wildtype, while exploring potential risk factors that may influence postoperative seizure control.

## Methods

### Patient population

A retrospective review was conducted on data from glioma patients who underwent surgical intervention at Beijing Tiantan Hospital between January 2015 and June 2021. The inclusion criteria were as follows: (1) age ≥ 18 years old; (2) diagnosed with GBM, IDH-wildtype after re-evaluation according to the 2021 WHO classification; (3) with sufficient demographic and clinical information. The exclusion criteria were as follows: (1) a history of anti-tumor treatment before the first surgery; (2) a history of other intracranial malignancies; (3) loss to follow-up; (4) having only received a biopsy. Demographic and clinical data were obtained from medical records retrieved from the electronic medical record system. The study was approved by the Ethics Committee of Beijing Tiantan Hospital, and informed consent was obtained from all patients or their relatives before surgery.

### Assessment for GRE

Our previous study provides a detailed explanation of the GRE assessment [11]. Preoperative GRE was defined as the occurrence of at least one unprovoked epileptic seizure prior to surgery, as determined by the patient’s primary complaints and medical history. For patients presenting with GRE, valproate or levetiracetam tablets were initiated or adjusted promptly following their clinical visits. All patients were administered prophylactic anti-seizure medications after surgery, which included phenobarbital injections provided within three days postoperatively, as well as valproate or levetiracetam tablets prescribed for a minimum duration of three months postoperatively. Seizure outcomes were longitudinally monitored through telephone interviews. If any unprovoked epileptic seizures occur within one year after surgery (the first surgery for newly diagnosed tumors, or the most recent surgery for recurrent tumors), the patient should be classified as having inadequate seizure control.

### Tumor evaluation

According to the 2021 WHO classification, GBM, IDH-wildtype was confirmed under the premise of an IDH-wildtype astrocytic glioma with any of the following features: (1) microvascular proliferation; (2) necrosis; (3) telomerase reverse transcriptase (TERT) promoter mutation; (4) epidermal growth factor receptor (EGFR) amplification; (5) a concomitant gain of chromosome 7 and loss of chromosome 10. The isocitrate dehydrogenase (IDH, IDH-wildtype was defined as the absence of detectable hotspot mutations, specifically targeting the R132 locus of IDH1 and the R172 locus of IDH2), O6-methylguanine-DNA methyltransferase (MGMT) promoter, and TERT promoter statuses were detected via pyrophosphate sequencing. The statuses of chromosome 7/10 and EGFR were identified through whole exome sequencing. All pathological diagnoses were independently reviewed and confirmed by two experienced neuropathologists. Any diagnostic discrepancies were resolved through consensus discussion with a third senior neuropathologist. Due to the limited number of tested patients (≤ two-thirds of the final cohort), only MGMT promoter status was included in the subsequent analysis.

The extent of resection (EOR) was assessed by comparing preoperative and postoperative magnetic resonance images obtained within 72 h after surgery, categorizing the results as gross total resection (GTR) or non-GTR. GTR was defined as no residual high-signal intensity on postoperative contrast-enhanced T1-weighted images. The EOR was independently evaluated by two experienced neurosurgeons, who were blinded to the clinical outcomes. In cases with inconsistent findings, a third senior neurosurgeon reviewed the cases to make a final determination.

### Clinical follow-up

Follow-up data were obtained through scheduled visits or telephone interviews. In addition to postoperative seizure occurrence, information on adjuvant therapy (whether treated with the Stupp protocol, i.e., radiation therapy combined with temozolomide), progression-free survival (PFS), and overall survival (OS) were also collected. PFS was defined as the duration from the date of surgery until that of radiographic progression, as identified according to the Response Assessment in Neuro-Oncology criteria, or until the date of the last follow-up. OS was defined as the duration from the date of surgery until that of death or the last follow-up.

### Statistical analysis

The IBM SPSS Statistics software (Version 26.0, IBM Corp., Armonk, New York, USA) and the GraphPad Prism software (Version 10.1.2, GraphPad Software Inc., San Diego, California, USA) were applied for data management and statistical analysis. Continuous variables were presented as mean values accompanied by corresponding standard deviations, and categorical variables were expressed as frequencies or proportions. Group comparisons were conducted utilizing the Chi-square test, Fisher’s exact test, Student’s t-test, or Mann-Whitney U-test according to the type of compared variables (continuous or categorical) and whether they followed a normal distribution (as determined by Kolmogorov-Smirnov test). The correlations of GRE with PFS and OS were confirmed by the Kaplan-Meier analysis and compared with the log-rank test. The effects were presented as hazard ratios (HRs) with 95% confidence intervals (CIs). Finally, multivariate binary logistic regression analysis was applied to explore potential risk factors for inadequate seizure control within one year after surgery. The goodness-of-fit of the model was evaluated using the Hosmer-Lemeshow test. Effects were reported as odds ratios (ORs) and corresponding 95% CIs. The level of statistical significance was set at *p* < 0.05.

## Results

### Patient characteristics

**Fig. 1:**
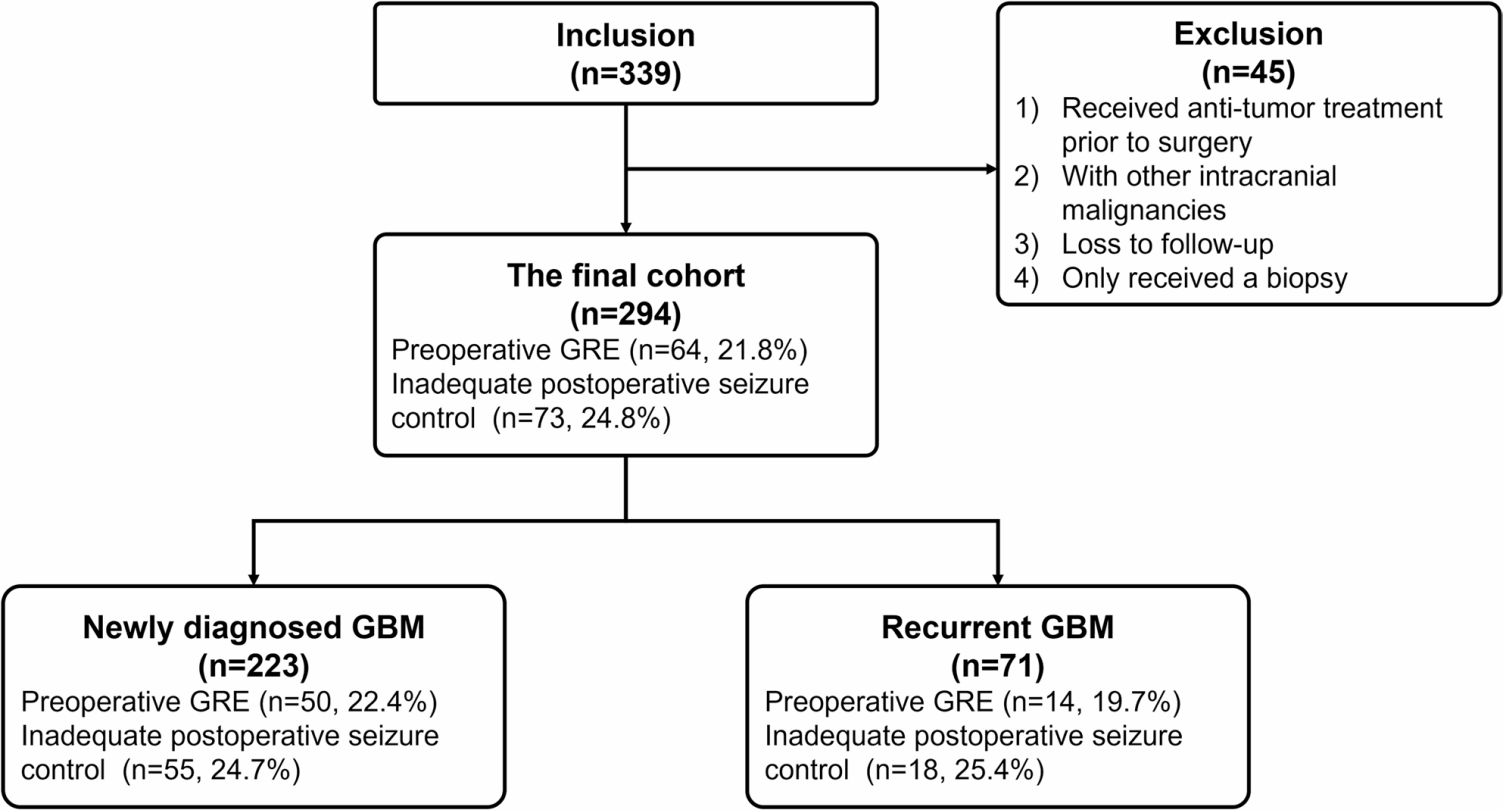
The flowchart of the current study. GRE, glioma-related epilepsy; GBM, glioblastoma

The final cohort comprised 294 patients diagnosed with GBM, IDH-wildtype. The flowchart is presented in Fig. 1. The mean age at diagnosis of the whole cohort was 51.60 ± 12.45 years, with a male-to-female ratio of 83:64. The incidence of preoperative GRE was 21.8% (64/294), and 73 individuals (24.8%) experienced seizure onset within one year after surgery.

Among the entire cohort, 223 patients had newly diagnosed GBMs, while 71 cases suffered recurrent tumors. There is no significant difference in the incidence rate of preoperative GRE between patients with newly diagnosed GBM and those with recurrent GBM (22.4% versus 19.7%, *p* = 0.631, Chi-square test). The rates of inadequate seizure control in the two groups were also comparable (24.7% versus 25.4%, *p* = 0.907, Chi-square test).

### The clinical correlations of preoperative GRE

**Fig. 2:**
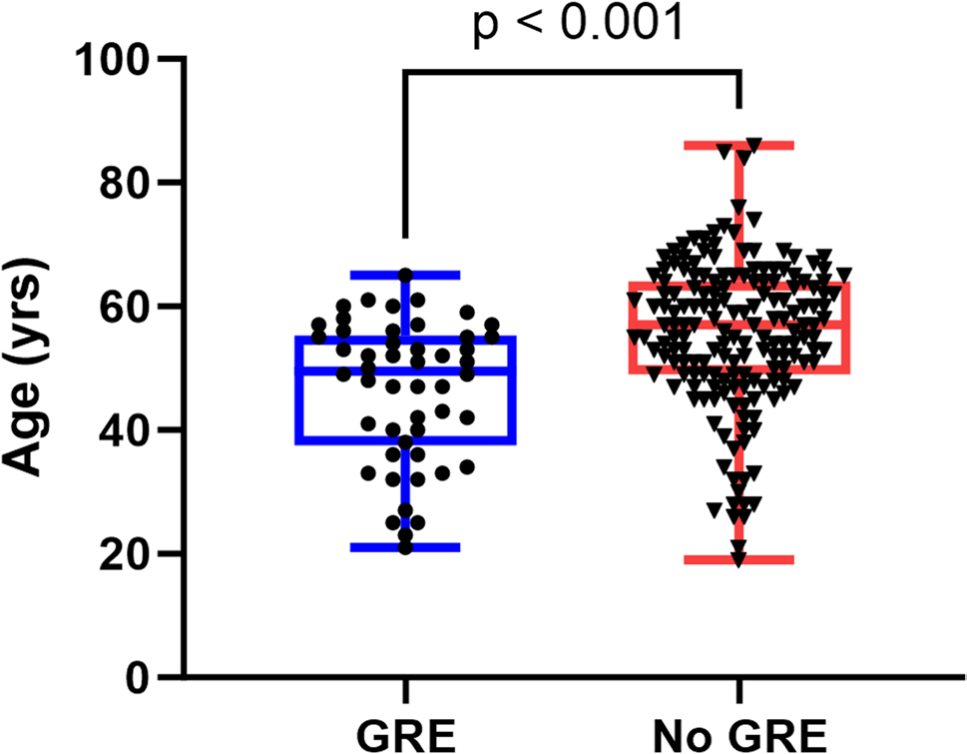
Age distribution comparison between patients with and without preoperative GRE in newly diagnosed GBMs. This box-and-whisker plot displays the age (in years) of patients with preoperative GRE (blue circles) and those without (red triangles). Boxes represent the interquartile range with the median line, and whiskers extend to the minimum and maximum values. A statistically significant difference in age distribution was observed between the two groups (*p* < 0.001, t-test). GBM, glioblastoma; GRE, glioma-related epilepsy

The clinical correlations of preoperative GRE in patients with newly diagnosed and recurrent GBM, IDH-wildtype are summarized in Table 1. Preoperative GRE was significantly correlated with younger age in patients with newly diagnosed GBM (*p* < 0.001, t-test, Fig. 2). No significant differences were found regarding gender, tumor side, tumor location, or preoperative Karnofsky Performance Score (KPS) between patients with preoperative GRE and those without it in this population. In patients with recurrent GBM, no correlations were demonstrated between preoperative GRE and any of the aforementioned variables. However, no matter whether in patients with newly diagnosed (*p* < 0.001, Chi-square test) or recurrent GBMs (*p* = 0.005, Fisher’s exact test), individuals with preoperative GRE were much more likely to experience seizures within one year after surgery.

**Table 1:** The correlations of preoperative GRE with other clinical characteristics in patients with newly diagnosed and recurrent GBM, IDH wildtype

Variables	Newly diagnosed (*n* = 223)			Recurrent (*n* = 71)		
	Preoperative GRE	No preoperative GRE	*p*-value	Preoperative GRE	No preoperative GRE	*p*-value
Number	50	173		14	57	
Age	46.46 ± 11.42	55.4 ± 11.95	< 0.001^a^	44.14 ± 10.70	46.39 ± 11.03	0.495^a^
Gender						
Male	33	90	0.080^b^	9	34	
Female	17	83		5	23	
Tumor side			0.556^b^			0.580^c^
Left	28	85		5	25	
Right	20	75		6	29	
Bilateral	2	13		3	3	
Temporal involvement			0.473^b^			0.953^b^
Yes	24	93		7	29	
No	26	80		7	28	
Preoperative KPS			0.185^b^			0.337^d^
≥80	47	151		12	53	
<80	3	22		2	4	
Extent of resection			0.373^b^			0.188^b^
GTR	19	78		5	11	
Non-GTR	31	95		9	46	
Postoperative KPS			0.114^b^			0.256^b^
≥80	37	107		6	34	
<80	13	66		8	23	
MGMT promotor			0.518^b^			1.000^d^
Methylated	17	76		5	14	
Unmethylated	18	63		6	21	
Treated with the Stupp protocol			0.340^b^			0.241^b^
Yes	39	111		4	24	
No	9	38		9	25	
Postoperative GRE within one year			< 0.001^b^			0.005^d^
Yes	27	28		8	10	
No	23	145		6	47	

*GRE* Glioma-related epilepsy, *GBM* Glioblastoma, *KPS* Karnofsky performance score, *GTR* Gross-total resection, *MGMT* O6-methylguanine-DNA methyltransferase

^a^Results of Student’s t-test

^b^Results of Chi-square test

^c^Results of Chi-square test with data combination

^d^Results of Fisher’s exact test

Notably, there was no significant difference in baseline variables that influence survival between patients with preoperative GRE and those without, including EOR, postoperative KPS, MGMT promoter status, and whether they were treated with the Stupp protocol. This baseline balance ensured that the subsequent survival differences between the two groups were not confounded by these factors.

### Survival analysis

**Fig. 3:**
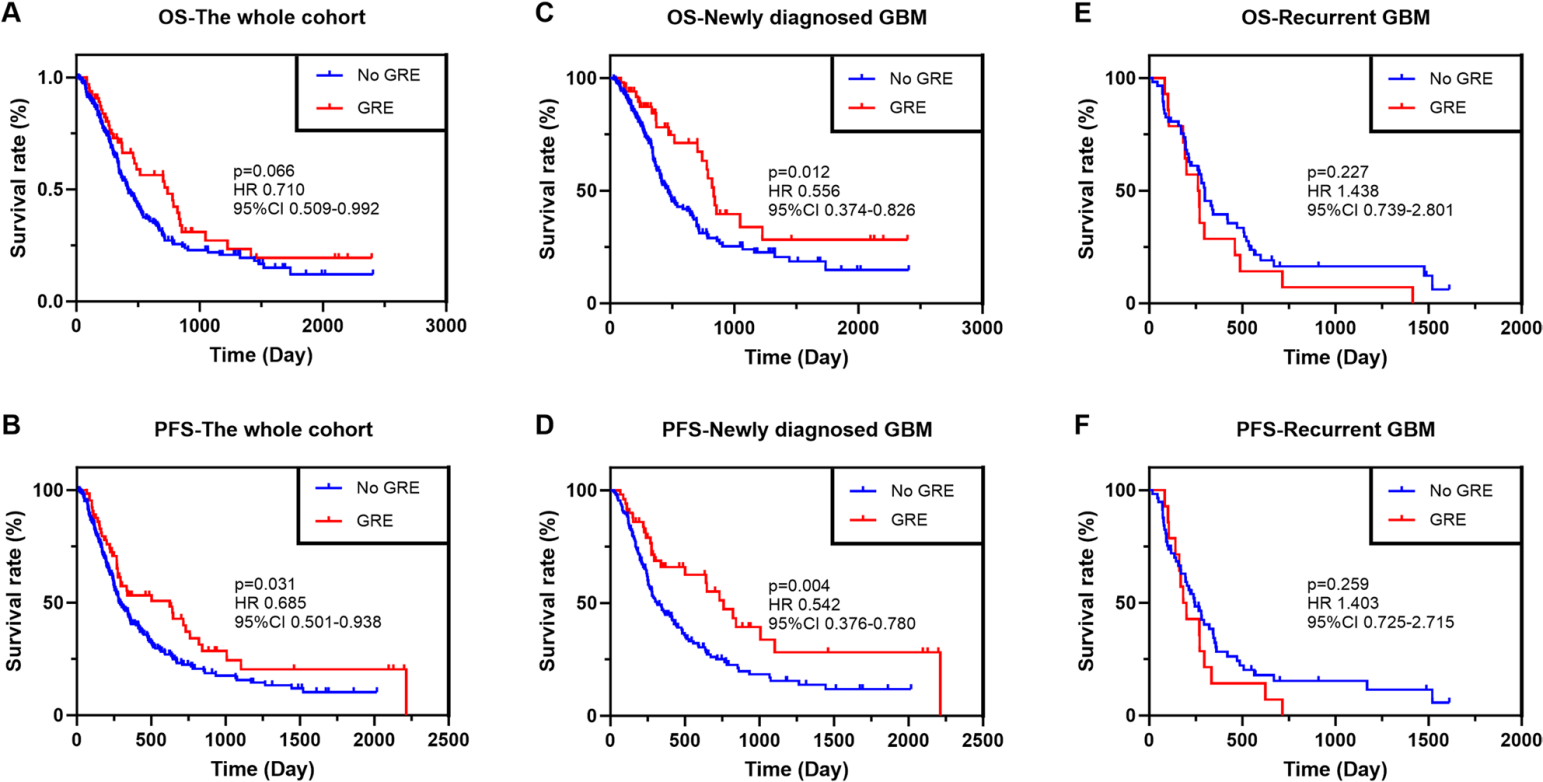
The comparisons of survival outcomes between patients with preoperative GRE and those without. The figure represents comparisons of (**A**) OS between the two subgroups in the whole cohort; (**B**) PFS between the two subgroups in the whole cohort; (**C**) OS between the two subgroups in patients with newly diagnosed GBM; (**D**) PFS between the two subgroups in patients with newly diagnosed GBM; (**E**) OS between the two subgroups in patients with recurrent GBM; (**F**) PFS between the two subgroups patients with recurrent GBM. The figure presents the HR (GRE/No GRE), 95% CI, and exact p-value for each log-rank test. GRE, glioma-related epilepsy; OS, overall survival; PFS, progression-free survival; GBM, glioblastoma; HR, hazard ratio; CI, confidence interval

Of the 294 enrolled patients, 186 cases (63.3%) died during the follow-up period. The relationship between preoperative GRE and survival outcomes was initially explored in the whole cohort. The results are shown in Figs. 3A and B. Preoperative GRE appeared to correlate with improved PFS (*p* = 0.031) but did not reach significance for OS (*p* = 0.066). Given that newly diagnosed and recurrent GBMs are already at different stages of the disease process, subgroup analyses were performed separately for each population. In patients with newly diagnosed GBM, preoperative GRE was associated with favorable OS (*p* = 0.012, Fig. 3C) and PFS (*p* = 0.004, Fig. 3D). Conversely, no predictive value for OS (*p* = 0.227, Fig. 3E) or PFS (*p* = 0.259, Fig. 3F) was observed in patients with recurrent GBM.

### Risk factors for inadequate postoperative seizure control

**Fig. 4:**
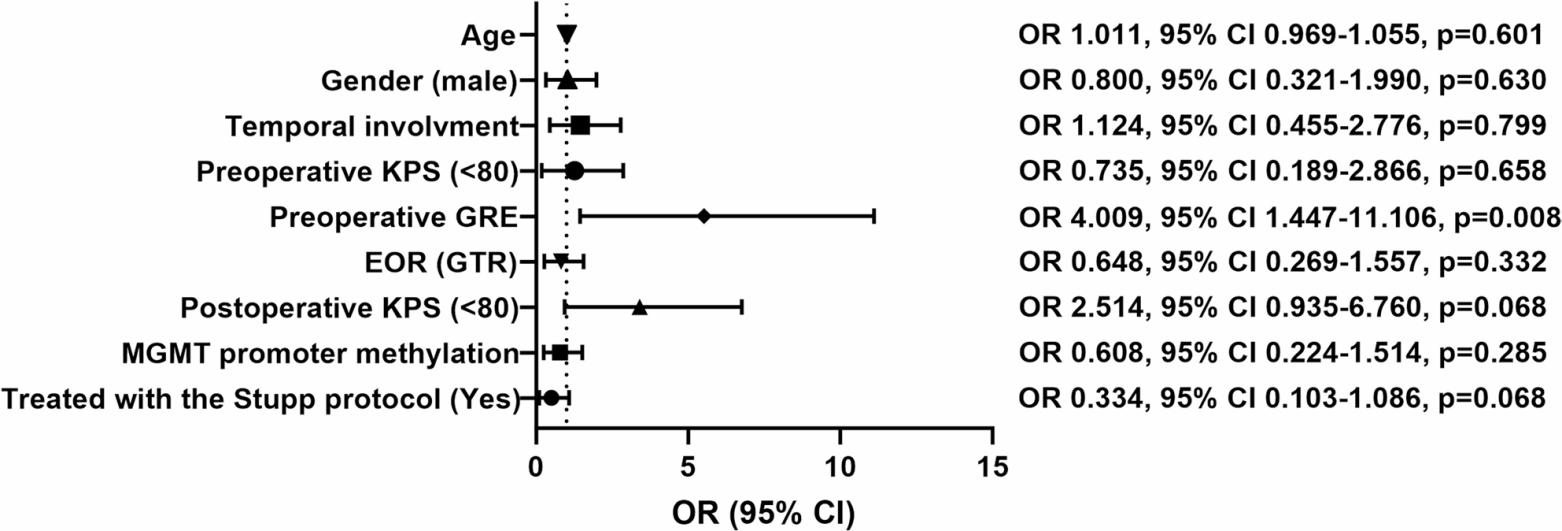
The result of multivariate binary logistic regression analysis with potential variables predicting postoperative seizure outcome one year after surgery. The figure presents the OR, 95% CI, and exact p-value for each variable. All 104 patients included in the model had complete data for all variables. OR, odds ratio; CI, confidence interval; KPS, Karnofsky performance score; GRE, glioma-related epilepsy; EOR, extent of resection; GTR, gross-total resection; MGMT, O6-methylguanine-DNA methyltransferase

Finally, the potential risk factors for inadequate postoperative seizure control within one year after surgery were explored through multivariate binary logistic regression analysis. To enhance the clinical relevance of the findings, additional patient screening was conducted. First, patients who lived without seizures but had a follow-up period of less than one year were excluded, as we could not assume they would remain seizure-free until one year after surgery. Second, given the critical role of the Stupp protocol and MGMT promoter methylation status in GBM management, patients who lacked relevant information were also excluded. Third, due to the limited sample size of the recurrent group, multivariate analysis was restricted to patients with newly diagnosed GBM. Ultimately, 104 cases were enrolled in the analysis, with the model accounting for variables including age, gender, temporal involvement, preoperative KPS, preoperative GRE history, EOR, postoperative KPS, MGMT promoter status, and the administration of the Stupp protocol. The result is shown in Fig. 4. Preoperative GRE was identified as the sole independent risk factor for inadequate postoperative seizure control within one year after surgery (OR 4.009, 95% CI 1.447–11.106, *p* = 0.008). The Hosmer-Lemeshow test for model goodness-of-fit yielded a p-value of 0.863, indicating that the model fit the data well, and its predictions were reliable.

## Discussion

In the 2016 WHO classification, GBM still includes both IDH-mutant and IDH-wildtype tumors. In the 2021 WHO classification, wild-type IDH has become a prerequisite for diagnosing GBM, and the term “GBM, IDH-wildtype” is now specifically applied to define this subset of adult-type diffuse gliomas. Compared to the 2016 classification, IDH-mutant tumors have been excluded from this subset, while part of the IDH-wildtype tumors may now be reclassified as pediatric-type diffuse gliomas or other types of CNS tumors. In addition, some previously diagnosed IDH-wildtype astrocytomas have been incorporated into the updated subset. In summary, significant changes have occurred in the category of GBM, and re-evaluating the clinical features of GRE under this new category holds considerable clinical and research value.

GBM represents the type with the lowest incidence of GRE among diffuse gliomas. According to previous literature, the incidence of preoperative GRE in GBM typically ranges from 25% to 50%, which is markedly lower than that observed in lower-grade gliomas [12–14]. Given the well-established positive association between IDH mutation and preoperative GRE, it can be anticipated that the incidence of preoperative seizures will further decrease in the newly classified subset of GBM, following the exclusion of IDH-wildtype tumors [7, 15]. In the current study, the incidence of preoperative GRE was 21.8% among a cohort of nearly 300 individuals, a rate comparable to that observed in meningiomas or metastatic tumors [16]. Moreover, no significant difference was observed in the incidence of GRE between newly diagnosed and recurrent tumors. We once described the characteristics of GRE in patients with high-grade gliomas under the 2016 WHO classification [17]. The findings indicated that the incidence of preoperative GRE in IDH-wildtype GBM was 20.0%, aligning well with the present finding. However, in two previously reported GBM cohorts classified according to the 2021 WHO classification, the incidences of preoperative GRE were 35.3% and 33.1%, which were significantly higher than in our cohort [18, 19]. We hypothesize that, in addition to the inter-center variation, the regional healthcare system attributes within China may also contribute substantially to this observed discrepancy. As GRE presents as a clinically apparent symptom, patients exhibiting preoperative GRE are typically identified and managed at local medical centers without the need for referral to a national-level tertiary institution such as ours. Furthermore, tumor molecular heterogeneity between our patients and those in prior studies may also contribute to the incidence difference, particularly for molecules that can modulate tumor epileptogenicity through mechanisms independent of IDH status, for instance, ion channel dysfunction [20, 21].

In addition, we revealed a statistically significant association between preoperative GRE and younger age in patients with newly diagnosed GBM, IDH-wildtype. This finding aligns with results from an independent GBM cohort classified according to the latest classification [18]. The primary influence on the young population is a common epidemiological feature of GRE among patients with adult-type diffuse gliomas [22, 23]. Historically, IDH mutation has often been explained as one of the underlying mechanisms for this association. Specifically, IDH-mutant gliomas are known to induce GRE more frequently and are typically diagnosed in younger patients, in contrast to IDH-wildtype tumors [15, 24]. The new classification further confirmed the connection between preoperative GRE and age while excluding the impact of IDH mutation status. The biological basis of this correlation remains incompletely understood and warrants further investigation. Variations in brain network organization across age groups may be a potential research direction. Younger individuals typically retain greater baseline brain network connectivity and neuroplasticity compared to older counterparts [25, 26]. Their preserved brain networks may be more susceptible to tumor-related microenvironmental changes that can trigger GRE, such as ion channel dysfunction, gap junction impairment, neurotransmitter imbalances, synaptic abnormalities, and neuroinflammation. In contrast, older patients often have preexisting brain network fragility, where aggressive GBM growth is more likely to directly damage their brain networks, which may reduce the likelihood of GRE development.

In addition to its relationship with age, the predictive value of preoperative GRE for survival outcomes has also been historically related to IDH mutation status. Numerous studies have confirmed a correlation between preoperative GRE and favorable survival outcomes [27, 28]. In 2018, we performed a meta-analysis on this topic and demonstrated that preoperative GRE was an independent predictor of better OS and PFS in either low-grade glioma or GBM [29]. However, in patients with IDH-wildtype GBM, recent studies have reported conflicting results concerning the relationship between preoperative GRE and survival outcomes [30–33]. In the current study, we identified a significant correlation between preoperative GRE and improved OS and PFS among patients with newly diagnosed GBM, IDH-wildtype. The other two studies, which focused on newly classified GBM, reported similar findings [18, 19]. In other words, almost all studies conducted on GBM, IDH-wildtype under the 2021 WHO classification have consistently shown the prognostic significance of preoperative GRE. Although this relationship does not involve IDH mutations, it can be understood through the concept of the epilepsy network. Highly invasive brain tumors can lead to severe impairment of brain networks, which constitute the basis for seizure generation and propagation. In contrast, tumors exhibiting a less aggressive growth pattern are more likely to preserve the critical network for the development of GRE [34]. This hypothesis may partially explain the low incidence of GRE in GBM and could also shed light on the association between GRE and improved survival outcomes. Moreover, the aforementioned correlation of preoperative GRE with younger age could also be an underlying mechanism for its prognostic value. Notably, it should be emphasized that the favorable correlation between preoperative GRE and survival represents a clinical observation, and it cannot be concluded that GRE exerts a direct protective effect on patient survival. The underlying molecular mechanisms driving this association warrant further investigation.

Postoperative management for GRE represents a long-term clinical challenge in glioma management. Despite the widespread use of prophylactic anti-seizure medications, seizure control in glioma patients remains inadequate [35]. Accordingly, previous literature and the resulting guidelines or expert consensus do not recommend routine prophylactic anti-seizure medications after surgery, regardless of whether the patient experienced GRE preoperatively [36, 37]. However, we do not consider that completely excluding the prophylactic application of anti-seizure medications would benefit patients. Distinguishing high-risk groups via reliable risk factors and providing personalized treatment should be more practical and feasible. In the current study, we tried to explore the risk factors for inadequate postoperative seizure control in patients with newly diagnosed GBM, IDH-wildtype. Preoperative GRE was identified as the only independent risk factor. This finding aligns with results from previous studies [17, 18]. The presence of preoperative GRE generally represents that the patient already has a mature epilepsy network, which can also lead to postoperative seizures if any potential epileptogenic foci are not removed during surgery. Interestingly, EOR showed no significant impact on postoperative seizure control, which was an unexpected finding for us. This discrepancy may be explained by the current definition of GTR in GBM surgery and the unique pathophysiological mechanisms underlying GRE. It is increasingly recognized that GRE is not primarily driven by the tumor mass itself, but rather originates from alterations in the peritumoral microenvironment and disruptions in epileptic networks [38]. Given that GTR in GBM surgery is typically defined as the complete removal of the contrast-enhancing region, it may be insufficient to modify the epileptogenic peritumoral microenvironment or disrupt established epileptic networks. The application of intraoperative electrocorticography monitoring may facilitate the resection of epileptogenic tumor tissue that does not exhibit detectable abnormalities on preoperative imaging modalities [39].

Some limitations of the current study should be addressed. One of the most significant limitations is that, due to the retrospective nature of the study, data that could have facilitated a deeper investigation into the potential mechanisms underlying GRE, including tumor-specific molecular alterations, functional magnetic resonance imaging, and electroencephalogram, were limited in availability. Moreover, we initially intended to conduct a subgroup analysis between histological and molecular GBMs, but failed due to the small sample size of patients with molecular GBM. At last, the single-center design of this study may introduce selection bias, potentially affecting the generalizability of our findings. Overall, the current understanding of GRE is still insufficient. More fundamental and clinical research is warranted to improve its management.

## Conclusion

The incidence of preoperative GRE in patients with GBM, IDH-wildtype is approximately 20%. Among patients with newly diagnosed GBM, IDH-wildtype, preoperative GRE is more likely to occur in younger individuals and is associated with prolonged OS and PFS. Additionally, a history of preoperative GRE may increase the risk of inadequate seizure control after surgery in this population.

## Data Availability

The data analyzed in this study are available in the Chinese Glioma Genome Atlas database (http://www.cgga.org.cn/) [40]. Information related to the GRE, the integrated diagnosis, and the latest follow-up has not been updated.

## Abbreviations

GBM: Glioblastoma
GRE: Glioma-related epilepsy
OS: Overall survival
PFS: Progression-free survival
CNS: Central nervous system
WHO: World Health Organization
TERT: Telomerase reverse transcriptase
EGFR: Epidermal growth factor receptor
IDH: Isocitrate dehydrogenase
MGMT: O6-methylguanine-DNA methyltransferase
EOR: Extent of resection
GTR: Gross total resection
CI: Confidence intervals
HR: harzard ratio
OR: Odds ratios
KPS: Karnofsky performance score
